# Effectiveness of a home-based cognitive behavioral program to manage concerns about falls in community-dwelling, frail older people: results of a randomized controlled trial

**DOI:** 10.1186/s12877-015-0177-y

**Published:** 2016-01-06

**Authors:** Tanja A. C. Dorresteijn, G. A. Rixt Zijlstra, Antonius W. Ambergen, Kim Delbaere, Johan W. S. Vlaeyen, Gertrudis I. J. M. Kempen

**Affiliations:** Department of Health Services Research – Focusing on Chronic Care and Ageing, CAPHRI School for Public Health and Primary Care, Maastricht University, P.O. Box 616, 6200 MD Maastricht, The Netherlands; Department of Methodology and Statistics, CAPHRI School for Public Health and Primary Care, Maastricht University, P.O. Box 616, 6200 MD Maastricht, The Netherlands; Neuroscience Research Australia, University of New South Wales, P.O. Box 1165, Randwick, NSW 2031 Australia; Research Group Health Psychology, University of Leuven, Tiensestraat 102, 3000 Leuven, Belgium; Department of Clinical Psychological Science, Maastricht University, P.O. Box 616, 6200 MD Maastricht, The Netherlands

**Keywords:** Fear of falling, Cognitive behavioral therapy, Accidental falls, Activity of daily life, Aged, Randomized controlled trial

## Abstract

**Background:**

Concerns about falls are common among older people. These concerns, also referred to as fear of falling, can have serious physical and psychosocial consequences, such as functional decline, increased risk of falls, activity restriction, and lower social participation. Although cognitive behavioral group programs to reduce concerns about falls are available, no home-based approaches for older people with health problems, who may not be able to attend such group programs are available yet. The aim of this study was to assess the effectiveness of a home-based cognitive behavioral program on concerns about falls, in frail, older people living in the community.

**Methods:**

In a randomized controlled trial in the Netherlands, 389 people aged 70 years and older, in fair or poor perceived health, who reported at least some concerns about falls and related activity avoidance were allocated to a control (*n* = 195) or intervention group (*n* = 194). The intervention was a home-based, cognitive behavioral program consisting of seven sessions including three home visits and four telephone contacts. The program aims to instill adaptive and realistic views about fall risks via cognitive restructuring and to increase activity and safe behavior using goal setting and action planning and was facilitated by community nurses. Control group participants received usual care. Outcomes at 5 and 12 months follow-up were concerns about falls, activity avoidance due to concerns about falls, disability and falls.

**Results:**

At 12 months, the intervention group showed significant lower levels of concerns about falls compared to the control group. Furthermore, significant reductions in activity avoidance, disability and indoor falls were identified in the intervention group compared with the control group. Effect sizes were small to medium. No significant difference in total number of falls was noted between the groups.

**Conclusions:**

The home-based, cognitive behavioral program significantly reduces concerns about falls, related activity avoidance, disability and indoor falls in community-living, frail older people. The program may prolong independent living and provides an alternative for those people who are not able or willing to attend group programs.

**Trial registration:**

ClinicalTrials.gov, NCT01358032. Registered 17 May 2011

## Background

Concerns about falls, also referred to as fear of falling, are common among older people living in the community, with a prevalence of about 50 % regardless of whether people experienced a recent fall [1, 2]. Negative consequences of concerns about falls include loss of balance confidence, social isolation, anxiety and symptoms of depression, avoidance of daily activities, physical frailty, falls, loss of independence, and institutionalization [1, 3–9]. It is therefore important for older people to manage their concerns about falls to maintain daily function and independence. Previous studies suggested that addressing factors in multiple domains may effectively reduce concerns about falls [10–13].

‘A Matter of Balance’ (AMB) is a multicomponent, cognitive behavioral, community-based program to reduce concerns about falls. Matching one’s activities to one’s physical abilities is a main element in the intervention. Through realistic and adaptive appraisal, the intervention aims to reduce concerns about falls and related activity avoidance *without* increasing falls. Previous studies in community-dwelling older people showed the effectiveness of the program in reducing concerns about falls and associated activity avoidance without increasing actual falls [14]. Additionally, the Dutch version of AMB (AMB-NL) demonstrated a reduction in the restriction of daily living activities and recurrent fallers [15]. The program uses cognitive restructuring and behavioral change techniques to address psychosocial (e.g., beliefs about falls and fall risk, social support, and assertiveness), physical (e.g., participation in physical activities and vision screening) and functional (e.g., safe behavior, participating in and continuing daily life activities) factors related to concerns about falls. AMB was originally developed as a group-based program, which can be delivered by trained healthcare professionals or volunteers. After evaluation research, the group program has been successfully implemented in different settings, versions and countries [16–21]. Despite the success of AMB, not all eligible older people participate. Especially those with health problems tend to withdraw prior and during the group program [14, 22]. In addition, not all older people prefer to participate in a group approach [23]. To allow particularly frail older people to participate in the program and to benefit from its effects an individualized, home-based format of AMB-NL additional to the group approach was therefore developed [24]. The home-based format of ‘A Matter of Balance’ (AMB-Home) includes three home visits and four telephone contacts and aims to encourage independent living among older people for as long as possible with minimal burden for healthcare professionals and informal caregivers [25–27].

The current paper reports on the results of a randomized controlled trial to evaluate the effects of AMB-Home compared with usual care on concerns about falls in community-dwelling, frail older people. Secondary outcomes of the trial were avoidance of activity due to concerns about falls, disability, and indoor and outdoor falls.

## Methods

### Study design

In this two-group randomized controlled trial (RCT), community-dwelling older people were selected in four consecutive cycles in 2009. In the Netherlands all citizens are registered in municipal registry offices and to select a representative sample, addresses of potential participants were randomly drawn by three offices in the south of the country. All cycles started between March and December 2009, and each cycle lasted 15 months. A cycle included screening for eligible participants, baseline measurements, stratified randomization, an intervention period of approximately 4 months, and two follow-up measurements at 5 and 12 months. To screen for eligibility, people received a short postal questionnaire with a freepost envelope, as well as information about the trial and an informed consent form. The Medical Ethics Committee of the Maastricht University/Academic Hospital Maastricht in the Netherlands approved the study (MEC 07-3-064). The trial was performed as planned. Additional information about the study design can be found in a published study protocol [24].

### Participants

Community-dwelling people aged 70 years or older were included in the study if they reported at least some concerns about falls and associated activity avoidance, perceived their general health as fair or poor, and were willing to participate (signed informed consent form) (see Table 1). The criteria with respect to concerns about falls and associated activity avoidance were based on two items: 1) “Are you concerned about falling?” and 2) “Do you avoid certain activities due to concerns about falling?” Answer options for both items included ‘never’ , ‘almost never’ , ‘sometimes’ , ‘regularly’ , ‘often’ , and ‘very often’. We included individuals with answers ranging from ‘sometimes’ to ‘very often’ regarding both concern about falling and activity avoidance. In this study, we used the term “frail” in relation to our sample to indicate that our sample perceived their general health as fair or poor in conjunction with reported concerns about falls and related activity avoidance. As a result we could make clear that we included those participants who were unlikely to participate in a group program because of health problems. Individuals were excluded if they were confined to bed; wheelchair dependent; waiting for nursing home admission; or experienced substantial hearing, vision or cognitive impairments. All inclusion and exclusion criteria were assessed during the screening, with the exception of cognitive impairment, which was assessed during the baseline measurement using the 4-item Abbreviated Mental Test (AMT4) [28]. If individuals scored <4 on the AMT4, the Telephone Interview Cognitive Status (TICS) was administered. Individuals were excluded if they scored <17 out of 41 on the TICS [29]. Additionally, a restriction was applied to couples; only one member of a couple was allowed to participate in the trial to prevent reciprocal influencing if by chance one was allocated to the treatment group and one to the control group. Lots were drawn to determine who of the couple would be included.

**Table 1:** Baseline Characteristics of Participants (*N* = 389)

	Control group (*n* = 195)		Intervention group (*n* = 194)		*p*-value
Demographic					
*Mean age in years (SD)*	78.25	(5.3)	78.38	(5.4)	0.81
*Gender (%)*					0.36
Male	54	(27.7)	62	(32.0)	
Female	141	(72.3)	132	(68.0)	
*Living situation (%)*					0.45
Not alone	77	(39.5)	84	(43.3)	
Alone	118	(60.5)	110	(56.7)	
*Educational level (%)*					0.20
Low	100	(51.5)	110	(57.3)	
Middle	72	(37.1)	55	(28.6)	
High	22	(11.3)	27	(14.1)	
Health-related					
*Perceived general health (%)*					0.16
Fair	176	(90.3)	166	(85.6)	
Poor	19	(9.7)	28	(14.4)	
*Mean number of active chronic diseases (SD)*	1.62	(1.0)	1.57	(1.0)	0.66
Fall-related					
*Falls in the past 6 months (%)*					0.11
Never	81	(42.2)	64	(33.3)	
Once	55	(28.6)	54	(28.1)	
More than once	56	(29.2)	74	(38.5)	
*Concerns about falls (%)*					1.00
Sometimes	90	(46.2)	91	(46.9)	
Regular	54	(27.7)	53	(27.3)	
Often	32	(16.4)	31	(16.0)	
Very often	19	(9.7)	19	(9.8)	
*Avoidance of activities (%)*					0.29
Sometimes	104	(53.3)	85	(43.8)	
Regular	50	(25.6)	62	(32.0)	
Often	25	(12.8)	27	(13.9)	
Very often	16	(8.2)	20	(10.3)	

Note: all numbers and percentages may not add up to final numbers due to missing data

### Randomization

To prevent an imbalance between groups, stratified randomization was used to randomly allocate participants to either the control group or the intervention group. A computerized two-block randomization was performed using the level of concerns about falls (i.e., sometimes, regular, often, and very often) as the prognostic factor. An external agency blinded to participant characteristics conducted the randomization directly after the baseline measurement. Cross-over between groups was not permitted, and participants were aware of their group allocation.

### Intervention

The purpose of our individual, home-based, cognitive behavioral AMB-Home program was to shift maladaptive to adaptive cognitions with respect to falling and concerns about falls. The program aims to instill a realistic view of fall risk, increasing self-efficacy beliefs and feelings of control, and changing behavior. To achieve these goals the following strategies were applied: 1) identifying and restructuring misconceptions about falls and fall risk; 2) setting realistic personal goals for increasing activity levels and safe behavior; and 3) promoting the uptake of old and new daily life activities that were avoided due to concerns about falls.

The AMB-Home program consists of seven individual sessions, including three home-visits (60, 60 and 75 min, respectively) and four telephone contacts (35 min each). The seven pre-defined themes of the program were concerns about falls; thoughts about falling; physical exercise; asserting oneself; overcoming personal barriers; safe behavior; and managing concerns about falls [24]. Each session was similarly structured with a review of the previous session (except the first session), a discussion of the main theme, and the formulation of a personalized action plan related to the discussed theme. Session 5 differed slightly from the other sessions in that participants were guided to safely execute a daily activity they were afraid to perform independently (‘exposure in vivo’) [30]. Examples of activities selected by participants included walking down the stairs or crossing a street. The participants received homework assignments between the sessions, including reading informative leaflets, filling in checklists to become aware of their beliefs about falls, and executing personal action plans. In addition, a DVD was used to show how peers address concerns about falls.

AMB-Home includes detailed manuals for both the participants and the program facilitators. The facilitators were community nurses (*n* = 8) who were qualified in the field of geriatrics and worked at local home-care agencies. Prior to the start of the trial, the nurses received a 2-day, mandatory training. During this training, the nurses became familiar with the content of the program and behavior change techniques. Professionals with expertise in motivational interviewing, behavioral change, and ‘exposure in vivo’ contributed to the training program.

Overall, principles for behavior change and themes of the group program were maintained in AMB-Home. However in adapting the group program to a home-based program several changes were made. First, the physical exercises in the group program were replaced by ‘exposure in vivo’ [30], because the appropriate and safe execution of these exercises could not be guaranteed due to the limited face-to-face contact. Second, motivational interviewing was incorporated to encourage internal motivation to change behavior and increase self-efficacy [31]. Next, participants were encouraged to invite a significant other (e.g., a spouse, friend, or neighbor) to be present during the home visits. This person could motivate the participant to perform the action plans between the sessions. Lastly, the eight group sessions (120 min each) were replaced by seven individual sessions, including three home-visits (60, 60 and 75 min, respectively) and four telephone contacts (35 min each). We considered that modeling and vicarious experiences are active ingredients of self-efficacy theory and are despite the use of the DVD less pronounced in the home-based program, individual support from the facilitator by action planning is more dominant in AMB-Home as well as the potential impact of a significant other. An overview of AMB-Home and the differences with AMB-NL are described elsewhere [24].

The control group received care as usual. Whereas no standard treatment for concerns about falls was available during the study period it is likely they received no treatment.

### Outcome measurements

With the exception of the registration of falls using a monthly calendar, data were collected at baseline and at 5- and 12-month follow-up via telephone interviews. Facilitators and participants were aware of group assignments; outcome assessors were blinded to the allocation. Prior to data collection, the outcome assessors from a center for data and information management participated in a 2-hour training session on assessment procedures and study design.

The primary outcome was concerns about falls measured with the 16-item Falls Efficacy Scale-International (FES-I; range 16 to 64). This scale assesses an individual’s level of concerns about falls while performing activities of daily living (ranging from 1 = not at all concerned to 4 = very concerned) [32, 33].

Secondary outcomes included avoidance of activity as a result of concerns about falls, disability, number of falls, and medical attention received after a fall incident. Avoidance of activity was measured using a modified version of the 16-item FES-I. If participants indicated that they experienced at least some concerns about falls while performing a certain activity, they were asked to indicate to what extent they avoided that activity as a result of their concerns (Falls Efficacy Scale-International Avoidance Behavior (FES-IAB); 1 = never and 4 = often; range 16 to 64) [24, 34]. Disability was measured using the 18-item Groningen Activity Restriction Scale (GARS). The GARS assesses the extent to which individuals have difficulty in performing 18 activities of daily living (ranging from 1 = yes, can do fully independently to 4 = no, can do only with help from others; range 18 to 72) [35]. Both disabilities in the areas of Activities of Daily Living, including mobility, (ADL; 11 items; range 11 tot 44) and Instrumental Activities of Daily Living (IADL; seven items; range 7 to 28) are embedded in the GARS. Falls were assessed using a monthly calendar that indicated whether a fall occurred in the past week. A fall was defined as an event that results in a person coming to rest inadvertently on the ground or on another lower level [36]. If a fall occurred, participants reported the location of the fall (indoor or outdoor) and the number of times medical attention related to the fall was received. Participants returned the calendar sheets each month and were reminded by telephone if a calendar was not returned after 10 days.

Demographic characteristics including age, gender, living situation, educational level (low: completed elementary school; middle: completed secondary school; high: completed higher vocational training or university level [37]), perceived general health, falls in the past 6 months, and active chronic diseases (i.e., diseases for which a physician was consulted or medicines were administered in the previous 12 months) were collected.

### Sample size

To detect a mean difference of at least 3.8 points (effect size of .33 on the FES-I) between the intervention and control group, 112 participants per group were required to provide 80 % power at alpha .05 (one-tailed). These sample size calculations were based on outcomes of a previous study using the FES-I among older people in the Netherlands [33]. However, we expected a 20 % dropout rate during the current study; therefore at least 280 (2 × 140) participants were needed for the final analyses in this trial [24].

### Statistical analyses

Descriptive techniques were used for the variables of interest. Data were analyzed according to the intention-to-treat principle; therefore, all participants were included based on their original allocation. Missing values were imputed at the level of the scale by means of multiple imputations. The maximum number of missing values within a scale was based on guidelines provided by the developers. A limit of 25 % missing values was used if no guidelines were available. Because multilevel analyses are quite robust against missing values at the measurement level, only the baseline measurement and one of the two follow-up measurements were needed to include participants in the analyses. The number of fallers, falls and fall-related medical attention were analyzed with negative binomial regression models and logistic regression models. All other outcomes were assessed using mixed-effects linear regression analyses. Models were adjusted for the stratification factor (i.e., concerns about falls), the baseline value of the outcome measure, age, gender, perceived general health, and number of falls in the 6 months before baseline. These covariates were considered a priori as relevant to the outcomes based on the literature [24]. The interaction term *group X time* was added to the model to determine the effects of the intervention (i.e., differences between the intervention and control group) at the two follow-up measurements. The level of statistical significance was set at .05 for those intervention effects where we expected an improvement in function (one-tailed), i.e., concerns about falls, avoidance of activities, and disability [24]. For the baseline characteristics and fall data, the level of statistical significance was set at .05 (two-tailed). The results are presented with adjusted mean differences, odds ratios (ORs) or incidence rate ratios (IRRs), 95 % confidence intervals (CIs) and, if applicable for significant differences, effect sizes. Effect sizes of .20 are considered small, .50 medium, and .80 large [38].

For the primary outcome the Reliable Change Index (RCI) score was computed to determine whether the change score (12-month follow-up score minus baseline score) of a participant lies outside the range of 95 % central change scores expected in case of no effect. The formula for RCI is change score divided by SE_diff_, where x_1_ represents a participant’s pretest score, x_2_ represents that same participant’s posttest score, SD_1_ is the standard deviation of the baseline scores and, *r* is the reliability of the measurement.

$$ \mathrm{R}\mathrm{C}\mathrm{I} = \kern0.5em \frac{x_2-{x}_1}{{\mathrm{SE}}_{\mathrm{diff}}}\kern0.5em \mathrm{and}\kern0.5em {\mathrm{SE}}_{\mathrm{diff}}\kern0.5em =\kern0.5em {\mathrm{SD}}_1\surd 2\surd \left(1-r\right) $$

The RCI is then compared with +/−1.96. The formula change score +/−1.96*SE_diff_ was used for a 95 % confidence interval for the true change scores [39, 40].

Furthermore, pre-planned per protocol analyses were performed; outcomes of participants in the control group were compared with those of intervention participants who received less than five sessions and intervention participants who received at least five of the seven program sessions [24]. Based on prior work, five sessions of the program were considered to be sufficient program exposure [14, 15]. All analyses were performed in SPSS 21.0.1 (SPSS, Inc., Chicago, IL).

## Results

### Participants

**Fig. 1:**
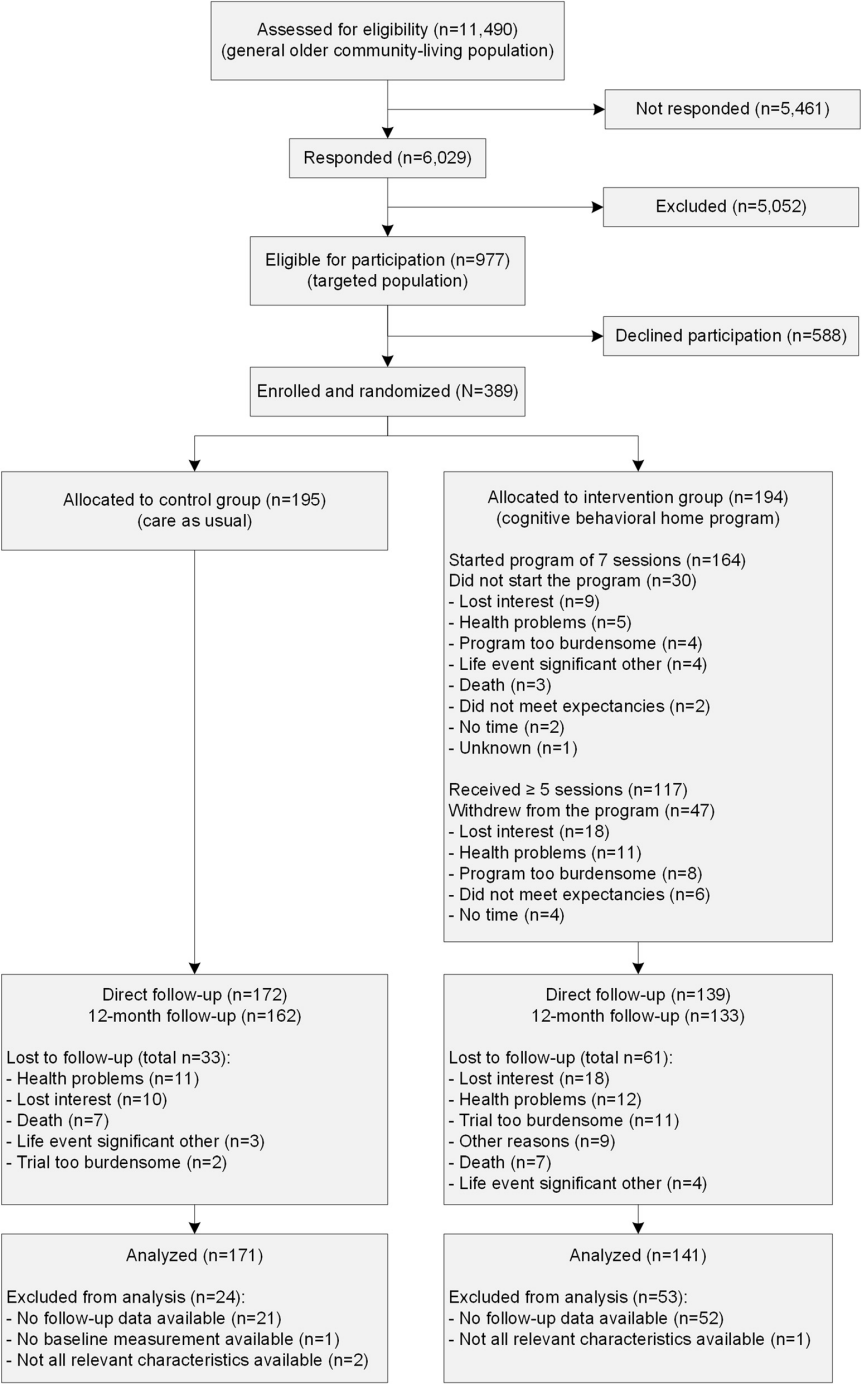
The flow of participants during the trial

The flow of participants during the trial is presented in Fig. 1. Eligibility screening occurred in the general community (see Study Design). Through randomization, 195 participants were allocated to the control group, and 194 participants were included in the intervention group. Baseline characteristics were comparable in both groups (Table 1). The dropout rates during the trial were 17 % (*n* = 33) in the control and 31 % (*n* = 61) in the intervention group. Withdrawal was highest at the 5-month follow-up measurement, which was directly after the intervention period. The main reasons for lost to follow-up were similar in the control and intervention group, i.e., lost interest and health problems. No significant differences were identified regarding baseline characteristics and primary and secondary outcomes between dropouts in the intervention and control groups (not tabulated). Thirty participants in the intervention group (15 %) were not exposed to the program; they withdrew from the intervention prior to the first session. Among the people who started the program, 29 % (47 out of 164) withdrew. Main reasons for not starting or withdrawal during the program were lost interest (*n* = 27), health problems (*n* = 16), and perceived burden (*n* = 8). Participants who received at least five of the seven sessions, rated their program engagement and satisfaction as high, and the burden of the program as low. More detailed information about the reach, fidelity, exposure, satisfaction and barriers of the program is published elsewhere [41].

### Outcomes

Table 2 indicates that the intervention significantly reduced concerns about falls at both the 5-month (adjusted mean difference = −3.53; *P* < .001) and 12-month follow-up (adjusted mean difference = −3.92; *P* < .001). Improvement was also observed for the secondary outcomes (Table 3), with the exception of IADL at the 5-month follow-up (adjusted mean difference = −0.52; *P* = .075). The effect sizes for significant differences were generally small to medium (.10 to .35).

**Table 2:** Effects of the Home-Based Cognitive Behavioral Program on Primary Outcome^a^

	Control group		Intervention group		Model^b^	*P*- value	Effect size
	*n* = 171		*n* = 141				
	Mean	(SD)	Mean	(SD)	Adjusted mean difference (95 % CI)	*P*	*d*
*FES-I*							
Baseline	35.47	(9.4)	35.70	(10.4)	-	-	-
5-month follow-up	35.30	(10.4)	31.73	(10.4)	−3.53 (−∞ – -2.15)	< .001	.34
12-month follow-up	35.86	(11.1)	31.98	(10.9)	−3.92 (−∞ – -2.52)	< .001	.35

Note: *FES-I* falls efficacy scale-international (range total scale 16–64; higher scores indicate more concerns about falls)

95 % CI = 95 % confidence interval (one-sided) *SD* standard deviation, ∞ = infinity

^a^Results of mixed-effects linear regression analyses (intention-to-treat)

^b^Adjusted for baseline score of the outcome and level of concerns about falls, age, gender, perceived general health, and falls in the past 6 months

**Table 3:** Effects of the Home-Based Cognitive Behavioral Program on Secondary Outcomes^a^

	Control group		Intervention group		Model^b^	*P*- value	Effect size
	*n* = 171		*n* = 141				
	Mean	(SD)	Mean	(SD)	Adjusted mean difference (95 % CI)	*P*	*d*
*FES-IAB*							
Baseline	29.09	(9.3)	29.06	(9.7)	-	-	-
5-month follow-up	28.74	(9.2)	26.17	(9.6)	−2.38 (−∞ − −1.12)	.001	.27
12-month follow-up	29.36	(10.3)	26.37	(10.4)	−2.67 (−∞ – −1.37)	.001	.29
*GARS*							
Baseline	33.73	(9.3)	34.11	(9.4)	-	-	-
5-month follow-up	33.32	(8.9)	32.42	(8.9)	−1.10 (−∞ – −0.07)	.040	.10
12-month follow-up	34.04	(9.3)	32.41	(9.4)	−1.81 (−∞ – −0.77)	.002	.17
*GARS ADL*							
Baseline	18.70	(4.9)	18.47	(4.9)	-	-	-
5-month follow-up	18.28	(4.5)	17.37	(4.7)	−0.62 (−∞ – −0.04)	.039	.20
12-month follow-up	18.69	(4.8)	17.60	(4.9)	−0.83 (−∞ – −0.24)	.011	.22
*GARS IADL*							
Baseline	15.03	(4.9)	15.64	(5.1)	-	-	-
5-month follow-up	15.05	(5.1)	15.04	(4.8)	−0.52 (−∞ – 0.08)	.075	-
12-month follow-up	15.35	(5.1)	14.82	(5.0)	−1.01 (−∞ – −0.41)	.003	.10

*FES-IAB* falls efficacy scale-international avoidance behavior (range total scale 16–64; higher scores indicate more activity avoidance due to concerns about falls)

*GARS* groningen activity restriction scale (range total scale 18–72; higher scores indicate more disability) *GARS ADL* groningen activity restriction scale — activities of daily living (ADL) subscale (range total scale 11–44; higher scores indicate more disability), *GARS IADL* groningen activity restriction scale—instrumental ADL subscale (range total scale 7–28; higher scores indicate more disability)

95 % CI = 95 % confidence interval (one-sided); SD = standard deviation; ∞ = infinity

^a^Results of mixed-effects linear regression analyses (intention-to-treat)

^b^Adjusted for baseline score of the outcome and level of concerns about falls, age, gender, perceived general health, and falls in the past 6 months

Table 4 shows the outcomes with respect to reliable change in concerns about falls. The SE_diff_ of the FES-I was 4.2. This means that the 95 % confidence interval of expected differences in case of no effect was between 8.2 and −8.2 points. Therefore a reliable improvement was defined as an improvement of at least 9 points on the FES-I between baseline and 12-month follow-up and a reliable deterioration was defined as a decrease of at least 9 points in this timeframe. Based on a RCI score of 1.96 or higher, 30 participants (22.6 %) improved in the intervention group versus 14 (8.7 %) in the control group. In addition, 23 participants (14.3 %) deteriorated in the control group versus 9 (6.8 %) in the intervention group (RCI score of −1.96 or lower).

**Table 4:** Reliable Change Index (RCI) of Concerns about Falls

	Control group		Intervention group	
	*n* = 161		*n* = 133	
*Concerns about Falls (FES-I)*	*n*	(%)	*n*	(%)
Reliable deterioration^a^	23	(14.3)	9	(6.8)
Not improved	124	(77.0)	94	(70.7)
Reliable improved^b^	14	(8.7)	30	(22.6)

Concerns about falls is measured with the FES-I (range total scale 16–64; higher scores indicate more concerns about falls). The FES-I reliable change index (RCI) score is calculated according to the outcomes on baseline and 12-month follow-up

^a^RCI score 1.96 or higher (equal to a FES-I score difference of 9 or higher)

^b^RCI score −1.96 or lower (equal to a FES-I score difference of −9 or lower)

The effects of the intervention on falls are presented in Table 5. Regarding the total number of falls, the number of outdoor falls and the number of times medical attention was required no significant differences were identified between the groups. Significantly fewer indoor falls were observed in the intervention group (IRR = 0.68; *P* < .014).

**Table 5:** Effects of the Home-Based Cognitive Behavioral Program on Fall Outcomes

	Control group		Intervention group		Model^a^	*P*- value
	*n* = 180		*n* = 166			
	*n*	(%)	*n*	(%)	OR (95 % CI)	*P*
*Fallers*						
Baseline until 12-month follow-up	106	(58.9)	94	(56.6)	0.79 (0.50–1.23)	.292
*Recurrent fallers*						
Baseline until 12-month follow-up	67	(37.2)	55	(33.1)	0.67 (0.41–1.09)	.104
	Number^b^		Number^b^		IRR (95 % CI)	*P*
*Total falls*	429		362		0.86 (0.65–1.13)	.273
Indoor falls	291		2		0.68 (0.50–0.92)	.014
Outdoor	138		160		1.11 (0.78–1.56)	.568
No. of times medical attention required as a result of falls	87		106		1.42 (0.96–2.10)	.083

Results of mixed-effects logistic and negative binomial regression analyses

95 % CI = 95 % confidence interval; OR = odds ratio mixed-effects logistics regression; IRR = incidence rate ratio obtained via negative binomial regression

^a^Model adjusted for baseline score measurement and level of concerns about falls, age, gender, perceived general health, and falls in the past 6 months

^b^Analyses were performed with a Poisson distribution. This distribution of fall events accounts for over dispersion and incorporates both number of falls and time (weeks) of follow-up; herefore, all available data was used

In contrast to the intention-to-treat analyses, a significant difference was identified in the per-protocol analyses for IADL at 5-months for those who participated in at least five sessions compared with the control group (adjusted mean difference = −0.64; *P* < .050). The effect sizes for the other outcomes (i.e., concerns about falls, related activity avoidance, and falls) remained similar (data not shown).

## Discussion

In this RCT the AMB-Home program significantly reduced concerns about falls in community-living, frail older people for up to 12 months. The home-based, cognitive behavioral program also showed favorable effects regarding the reduction of avoidance of activity due to concerns about falls, disability, and the number of indoor falls in the intervention group compared with the control group. No significant difference was found for total falls.

The outcomes of our study add to the increasing evidence that ‘A Matter of Balance’ as a cognitive behavioral approach positively influences concerns about falls and related avoidance behavior in older people. The setting and format in which the program is performed hardly affect the outcomes of the program, at least in the US and Western Europe [14, 15, 17, 18, 20, 21]. This facilitates the use of the program as it can be tailored to the preferences and abilities of older people [23].

Strengths of this study include a solid methodological design with a 1 year follow up period for the effect evaluation and a comprehensive process evaluation during the intervention period [24]. Recruitment of participants went as planned; a sample of frail people was selected from the general older population living in the community when we compare our sample with samples in previous trials [15, 42].

Some limitations are also recognized. First, in our study, a placebo group was not included to control for contact time and attention due to lack of financial and human resources. However, given the seriousness of the problem, it is unlikely that concerns about falls were significantly reduced exclusively by social elements [14]. Second, participants were not blinded; thus, they were aware of their group allocation, potentially introducing bias. Thirdly, our final follow-up assessment was conducted 12 months after baseline but only 7 months post intervention. This may hamper comparisons with other studies that applied a more common follow-up period of 12 months after the intervention. Our 7-month follow-up was guided by practical reasons (e.g., project finances) and comparability of effectiveness with AMB-NL. Lastly, dropout of study participants was substantial and different in both groups, i.e. 17 % in the control group and 34 % in the intervention group. Therefore selective dropout may be an issue although additional analyses showed no significant differences on selected baseline characteristics including primary and secondary outcomes between those who completed the trial and those who did not in the two study groups.

Before the dissemination of AMB-Home on a larger scale, some changes may be considered given the experiences during this trial and a simultaneous process evaluation [41]. First, the dropout rate in the intervention was considerable and a more effective and suitable procedure to screen older people is required for practice. A personal screening approach is recommended. During the nationwide implementation of the AMB-NL group program in the Netherlands this approach was applied and dropout rates reduced (from 42 % dropout in the trial to 17 % dropout in the implementation study) [15, 21]. A face-to-face intake procedure (e.g., in general practitioner practices, falls clinics, or by nurses of home-care organizations) can simultaneously clarify the suitability and preference of potential participants of either program format, i.e. group-based or a home-based, for the older person [23]. Second, improvements in compliance may add value to the program. The process evaluation [41] indicated that the use of action plans decreased towards the end of the program; the use ranged from more than 70 % in the first sessions to 51 % in the latter sessions. Additionally, the ‘exposure in vivo’ exercise (i.e., performing an activity safely under supervision of the facilitator) was only performed by half of the participants. Difficulty in finding an appropriate activity was given as main reason by facilitators for not performing an activity together with the participant. It had been foreseen that selecting suitable activities for the goal-setting and action-planning components of the program could be challenging [43]. To overcome this difficulty, 16 pictures of the Icon-FES [44], which include the daily activities used in the assessment of concerns about falls by the FES-I [32], were incorporated in session 2 and served as examples for the selection of activities by the participant., Providing more attention to goal setting and the execution of personal action plans in the later sessions is needed to increase the program compliance and this will presumable achieve stronger program effects. Goal setting and behavioral practice are considered as the most promising behavioral change techniques in AMB-NL [45].

Future studies may focus on defining the clinical relevance of the intervention effects, given that no criterion standards exist for levels of concerns about falls. Delbaere and colleagues [8] suggested a cut-off point for the Falls Efficacy Scale-International; however, more research is needed on this relevant subject as meaningful cut-off scores may vary across different samples and settings [46]. Also for the secondary outcomes no clinical relevant differences are known. In our study we have focused on meaningful changes according to RCI-scores and effect sizes. The outcomes indicate small to medium program effects. Nevertheless, small statistical effects may have substantial impact on daily life [47]. This is confirmed by the self-perceived benefits of the participants of the program [41]. Another focus is the program’s cost-effectiveness (in progress for ABM-Home) and the impact of individualizing the number of sessions, i.e., fitting the number of sessions to the anticipated effects regarding knowledge, skills and behavior. The latter would likely lead to a more cost-effective intervention. Future research may also focus on locating the most effective components within such complex interventions (e.g., education, action plans or ‘exposure in vivo’) and the effects of providing more attention to these components [45].

## Conclusions

In summary, AMB-Home reduced concerns about falls and associated avoidance of activity, as well as more downstream outcomes, such as disability and indoor falls in frail older people. The observed effects were small to medium, yet, present in a frail population and over a timeframe of 12-months. Therefore, this home-based, individualized AMB format is a welcome addition to current geriatrics care, particularly for those persons who are not able or willing to attend group programs. Future geriatric research should focus on improving participants’ and facilitators’ compliance, and on determining the components that are essential to achieve an increase in program effects in this older population.

## Abbreviations

AMB-Home: the Dutch in-home version of a matter of balance
AMB-NL: the Dutch group version of a matter of balance
