# Erratum to: Effectiveness of a home-based cognitive behavioral program to manage concerns about falls in community-dwelling, frail older people: results of a randomized controlled trial

**DOI:** 10.1186/s12877-016-0278-2

**Published:** 2016-05-24

**Authors:** Tanja A. C. Dorresteijn, G. A. Rixt Zijlstra, Antonius W. Ambergen, Kim Delbaere, Johan W. S. Vlaeyen, Gertrudis I. J. M. Kempen

**Affiliations:** Department of Health Services Research – Focusing on Chronic Care and Ageing, CAPHRI School for Public Health and Primary Care, Maastricht University, P.O. Box 616, Maastricht, MD 6200 The Netherlands; Department of Methodology and Statistics, CAPHRI School for Public Health and Primary Care, Maastricht University, Maastricht, The Netherlands; Neuroscience Research Australia, University of New South Wales, Barker St, New South Wales, Australia; Research Group Health Psychology, University of Leuven, Leuven, Belgium; Department of Clinical Psychological Science, Maastricht University, Maastricht, The Netherlands

## Erratum

Unfortunately, the original version of this article [1] contains an error within Table 5 of the results section. Within the column “Intervention group” the number of “Indoor falls” was incorrectly written as 2, but should in fact be 202. The correct version of Table 5 can be found below.

**Table 5:** Effects of the Home-Based Cognitive Behavioral Program on Fall Outcomes

	Control group		Intervention group		Model^a^	*P*- value
	*n* = 180		*n* = 166			
	*n*	(%)	*n*	(%)	OR (95 % CI)	*P*
*Fallers*						
Baseline until 12-month follow-up	106	(58.9)	94	(56.6)	0.79 (0.50-1.23)	.292
*Recurrent fallers*						
Baseline until 12-month follow-up	67	(37.2)	55	(33.1)	0.67 (0.41-1.09)	.104
	Number^b^		Number^b^		IRR (95 % CI)	*P*
*Total falls*	429		362		0.86 (0.65-1.13)	.273
Indoor falls	291		202		0.68 (0.50-0.92)	.014
Outdoor	138		160		1.11 (0.78-1.56)	.568
No. of times medical attention required as a result of falls	87		106		1.42 (0.96-2.10)	.083

Results of mixed-effects logistic and negative binomial regression analyses

95 % CI = 95 % confidence interval; OR = odds ratio mixed-effects logistics regression; IRR = incidence rate ratio obtained via negative binomial regression

^a^Model adjusted for baseline score measurement and level of concerns about falls, age, gender, perceived general health, and falls in the past 6 months

^b^Analyses were performed with a Poisson distribution. This distribution of fall events accounts for over dispersion and incorporates both number of falls and time (weeks) of follow-up; herefore, all available data was used
